# Patient-Reported Outcomes With Episoft® AC SPF 50+, a Ceramide-Based Moisturizer, in Acne-Related Post-procedural Care: A Real-World Multicenter Observational Study From India (PROCEED Study)

**DOI:** 10.7759/cureus.116524

**Published:** 2026-09-19

**Authors:** Gaurav Nakra, Maitreyee Panda, Soumya Jagadeesan, Sama Rais, Ashwin Balasubramanian, Prajakta Bhosale, Dhiraj Dhoot, Saiprasad Patil

**Affiliations:** 1 Dermatology, Centre for Skin, New Delhi, IND; 2 Dermatology, Institute of Medical Sciences and SUM Hospital, Bhubaneswar, IND; 3 Dermatology, Amrita Institute of Medical Sciences and Research Centre, Kochi, IND; 4 Dermatology, DERMA HUB Clinic, Mumbai, IND; 5 Global Medical Affairs, Glenmark Pharmaceuticals Limited, Mumbai, IND

**Keywords:** acne, barrier repair, ceramide, chemical peel, india, observational study, patient-reported outcomes, post-procedural skincare, spf 50+

## Abstract

Background: Post-procedural skin barrier disruption following acne-related dermatological procedures causes significant patient discomfort and heightens ultraviolet (UV) sensitivity. Ceramide-based formulations with broad-spectrum photoprotection can address barrier repair and sun protection factor (SPF) in a single step. However, patient-reported evidence supporting their use in this setting is still limited.

Methods: A retrospective, multicenter, observational study was conducted across 477 dermatology centers in India after ethics approval. Patients aged 18-45 years prescribed Episoft® AC SPF 50+ (a ceramide-based moisturizer with broad-spectrum SPF 50+ photoprotection; Glenmark Pharmaceuticals Limited, Mumbai, India) for at least seven days following acne-related dermatological procedures were included. Post-procedural skin barrier disruption was classified into four levels (none/minimal, mild, moderate, severe) based on procedure type. Patient-reported outcome measures (PROMs) were assessed at Day 7 using 10-point numeric scales. Primary endpoints were reduction in dryness and burning/stinging sensation. Secondary endpoints were reduction in skin itching, reduction in skin redness, soothing effect, acne worsening, overall satisfaction, willingness to continue use, and willingness for future procedures. Improvement responses were categorized as complete (9-10), moderate (6-8), mild (3-5), or none (1-2). Acne worsening used a reversed scale, in which a score of 1-2 indicated no/minimal worsening (favorable).

Results: A total of 2,066 patients (55.3% (n = 1,143) female; modal age group 26-30 years, 42.7% (n = 882)) were enrolled. The top three procedures were chemical peel (56.7%; n = 1,172), Q-switched laser toning (10.9%; n = 226), and microneedling (8.9%; n = 183). Barrier disruption was predominantly moderate (56.7%; n = 1,172), with severe disruption in 10.8% (n = 223). On Day 7, complete improvement was reported by 61.2% (n = 1,265) for dryness (mean ± SD: 8.64 ± 1.14) and 58.6% (n = 1,210) for burning/stinging (mean ± SD: 8.63 ± 1.14). Complete improvement rates for secondary endpoints were skin itching 61.4% (n = 1,268), skin redness 62.1% (n = 1,283), and soothing effect 61.2% (n = 1,264). Overall extreme satisfaction was reported by 62.6% (n = 1,293), willingness to continue use by 65.6% (n = 1,356), and willingness for future procedures by 67.2% (n = 1,388). No worsening of acne was reported by 67.9% (n = 1,403) of patients. There were no adverse events reported during the study.

Conclusion: In this large, real-world multicenter study, Episoft® AC SPF 50+, a ceramide-based moisturizer with SPF 50+, was associated with substantial patient-reported symptom relief, high satisfaction, and minimal acne worsening across diverse acne-related dermatological procedures and barrier severity levels, along with an excellent safety profile. These findings, together with emerging patient-reported outcome-based evidence for combined moisturizer-plus-SPF products, support routine inclusion of ceramide-based SPF 50+ moisturization in post-procedural skincare regimens for acne-related procedures.

## Introduction

Post-procedural skin barrier disruption is an expected and clinically significant consequence of acne-related dermatological procedures. Chemical exfoliation with agents such as salicylic and glycolic acid, microneedling, and energy-based modalities all induce intentional, controlled cutaneous injury, resulting in a transient period of compromised barrier integrity, heightened transepidermal water loss (TEWL), erythema, dryness, and increased ultraviolet (UV) sensitivity [1,2]. In patients with acne-prone skin, this post-procedural window carries additional clinical risks: barrier disruption may trigger acne flares, and the associated photosensitization necessitates effective sun protection to prevent post-inflammatory hyperpigmentation (PIH) in darker phototypes, particularly the Fitzpatrick III-V skin types that predominate in the Indian population [3,4].

Post-procedural skincare therefore needs to address two key needs: barrier restoration and photoprotection. Ceramide-based moisturizers are particularly suited for the first role. Ceramides are a critical lipid component of the stratum corneum and are essential for maintaining barrier cohesion, regulating TEWL, and supporting the lamellar body secretion pathway that reconstitutes the epidermal lipid matrix following injury [1]. Formulations combining ceramide replenishment with broad-spectrum photoprotection simultaneously address both the physical and photobiological effects of barrier disruption, offering a simplified skincare approach that may improve patient adherence during the post-procedural recovery period [3,5]. Despite this strong theoretical rationale, patient-reported evidence specifically evaluating their use in the context of acne-related procedures remains limited [2].

The growing volume of acne-related procedures performed across Indian dermatology clinics underscores the need for outcome data from this population. Chemical peels remain the most performed procedure for acne and PIH in Fitzpatrick skin types III-V, and Q-switched laser toning, microneedling, and radiofrequency microneedling are increasingly used as adjunctive and stand-alone treatments for acne scarring and active disease [6,3]. Across these modalities, periprocedural skincare practices vary considerably among practitioners, and large, real-world observational datasets capturing patient-reported outcome measures (PROMs) across multiple procedure types and barrier severity levels provide a useful complement to controlled trial evidence.

Hence, this study was conducted with the primary objective of evaluating patient-reported improvement ratings in post-procedural dryness and burning/stinging sensation on Day 7 following acne-related dermatological procedures treated with Episoft® AC SPF 50+ (Glenmark Pharmaceuticals Limited, Mumbai, India). Secondary objectives were to evaluate patient-reported improvement ratings in skin itching, skin redness, soothing effect/comfort, acne worsening, cosmetic elegance, overall treatment satisfaction, willingness to continue use, and willingness to undergo future procedures, and to evaluate outcomes across diverse procedure types and barrier disruption severity tiers. We hypothesized that combining ceramide-based barrier repair with broad-spectrum SPF 50+ photoprotection in a single daily formulation would provide substantial patient-reported symptom relief, high treatment satisfaction, and low rates of acne worsening across varying levels of procedural barrier disruption.

## Materials and methods

This was a multicenter, retrospective observational study conducted across 477 dermatology centers in India. As this was a retrospective analysis of anonymized medical records from routine clinical practice, individual patient consent was not required. The study adhered to the principles of the Declaration of Helsinki, complied with applicable Indian regulatory requirements including the New Drugs and Clinical Trials Rules, 2019 [7], and received central ethics approval before study initiation (approval dated 15 September 2025). At the scheduled Day 7 post-procedure follow-up visit, patients completed the standardized PROM questionnaire in clinic. Completed clinical and PROM records were subsequently compiled for centralized analysis.

Patients aged 18-45 years who had been prescribed Episoft® AC SPF 50+ (a ceramide-based moisturizer with broad-spectrum photoprotection) for at least seven days following an acne-related dermatological procedure were included. In accordance with real-world observational study design principles aimed at maximizing generalizability to routine clinical practice across India, no restrictive exclusion criteria were imposed beyond the requirement for complete clinical and PROM records.

Post-procedural skin barrier disruption was classified into four severity levels based on procedure type, adapted from established periprocedural skincare guidelines (Table 1) [2]. Barrier disruption level was also independently evaluated and categorized by treating dermatologists for each patient based on procedure intensity, depth of injury, energy density/fluence, number of passes, acid concentration/peel depth, and observed immediate cutaneous response (Appendix 1).

**Table 1:** Post-procedural Skin Barrier Disruption Classification by Procedure Type †Chemical peels are not energy-based devices; classified by clinical convention. Adapted from Goodman et al. 2025 [2]. CO2, carbon dioxide; Er:YAG, erbium-doped yttrium aluminum garnet; HIFU, high-intensity focused ultrasound; LED, light-emitting diode; YSGG, yttrium scandium gallium garnet.

Barrier Disruption Level	Procedures
None / Minimal	Q-switched laser toning (low energy), intense pulsed light (IPL), LED therapy (Red/Blue)
Mild	HIFU, radiofrequency microneedling, picosecond laser, non-ablative fractional laser (1,927 nm, low-energy density)
Moderate	Chemical peel (salicylic/glycolic acid)†, non-ablative fractional laser (1,927 nm, high-energy density), ablative radiofrequency, fractional ablative lasers
Severe	Fractional/full-field ablative resurfacing (Er:YAG, CO₂, YSGG)

PROMs were assessed on Day 7 using a 10-point numeric scale. Primary endpoints were improvement ratings for dryness at Day 7 and burning and/or stinging sensation at Day 7. Secondary endpoints were improvement ratings in skin itching, skin redness, soothing effect, worsening of acne, overall satisfaction, cosmetic elegance, willingness to continue use of Episoft® AC SPF 50+, and willingness for future procedures. Improvement ratings were categorized as complete (9-10), moderate (6-8), mild (3-5), or none (1-2). For acne worsening, a reversed 10-point scale was used, where 1-2 represented no/minimal worsening (favorable), 3-5 represented mild worsening, 6-8 represented moderate worsening, and 9-10 represented high/severe worsening. Any incidence of adverse events was also noted.

Descriptive statistics including integer frequency counts, percentages, and means ± standard deviations (SD) were calculated for all endpoints for the whole cohort and stratified by barrier disruption severity level and procedure type. Differences in continuous PROM ratings across barrier disruption severity strata were evaluated using the non-parametric Kruskal-Wallis H test and one-way analysis of variance (ANOVA). All analyses were conducted on complete verified source records with a two-sided significance threshold of p < 0.05.

The case record form used for data collection is provided in Appendix 2. The exact questions asked can also be found within it.

## Results

A total of 2,066 patients were enrolled across 477 dermatology centers. The cohort had 55.3% females, with age recorded in four groups (18-25, 26-30, 31-40, and 41-45 years); the 26-30-year group was the most common (42.7%). Chemical peel (salicylic/glycolic acid) was the most frequently performed procedure (56.7%; n = 1,172), followed by Q-switched laser toning (10.9%; n = 226), microneedling (8.9%; n = 183), radiofrequency microneedling (5.2%; n = 108), light-emitting diode (LED) therapy (4.9%; n = 101), picosecond laser (4.5%; n = 93), non-ablative fractional laser 1,927 nm (4.1%; n = 84), intense pulsed light (2.5%; n = 51), and high-intensity focused ultrasound (HIFU) (2.3%; n = 48).

Barrier disruption was predominantly moderate (56.7%; n = 1,172). Mild disruption accounted for 29.1% (n = 602), severe for 10.8% (n = 223), and none/minimal for 3.3% (n = 69).

At Day 7, 61.2% (n = 1,265) of patients reported complete improvement (score 9-10) for dryness (mean ± SD: 8.64 ± 1.14) and 58.6% (n = 1,210) for burning/stinging (mean ± SD: 8.63 ± 1.14). Moderate improvement (6-8) was reported by 36.5% (n = 755) for dryness and 39.5% (n = 816) for burning/stinging, and mild or no improvement remained low for both endpoints (dryness: 2.2% (n = 46); burning/stinging: 1.9% (n = 40); Table 2). The full response distribution is presented in Table 2.

**Table 2:** Patient-Reported Outcome Measures at Day 7 (Whole Cohort, N = 2,066) * - Primary endpoints. †For acne worsening, scoring was on a reversed scale: 1-2 = no/minimal worsening (favorable), 3-5 = mild worsening, 6-8 = moderate worsening, 9-10 = high/severe worsening. SD: standard deviation.

Outcome	Complete 9-10, n (%)	Moderate 6-8, n (%)	Mild 3-5, n (%)	None 1-2, n (%)	Mean	SD
PRIMARY ENDPOINTS						
Dryness improvement rating*	1,265 (61.2)	755 (36.5)	46 (2.2)	0 (0.0)	8.64	1.14
Burning/stinging improvement rating*	1,210 (58.6)	816 (39.5)	40 (1.9)	0 (0.0)	8.63	1.14
SECONDARY ENDPOINTS						
Skin itching improvement rating	1,268 (61.4)	759 (36.7)	39 (1.9)	0 (0.0)	8.69	1.11
Skin redness improvement rating	1,283 (62.1)	743 (36.0)	40 (1.9)	0 (0.0)	8.69	1.13
Soothing effect rating	1,264 (61.2)	761 (36.8)	39 (1.9)	2 (0.1)	8.70	1.12
Cosmetic elegance rating	1,331 (64.4)	694 (33.6)	39 (1.9)	2 (0.1)	8.75	1.09
Overall satisfaction rating	1,293 (62.6)	734 (35.5)	38 (1.8)	1 (0.0)	8.74	1.11
Willingness to continue use	1,356 (65.6)	668 (32.3)	42 (2.0)	0 (0.0)	8.77	1.11
Willingness for future procedure	1,388 (67.2)	642 (31.1)	35 (1.7)	1 (0.0)	8.84	1.13
ACNE WORSENING (reversed scale: 1-2 = favorable)						
Acne worsening†	268 (13.0)	257 (12.4)	139 (6.7)	1,402 (67.9)	3.28	3.16

Among secondary endpoints, 61.4% (n = 1,268) reported complete improvement in skin itching (mean ± SD: 8.69 ± 1.11), 62.1% (n = 1,283) in skin redness (mean ± SD: 8.69 ± 1.13), and 61.2% (n = 1,264) in soothing effect (mean ± SD: 8.70 ± 1.12). Overall extreme satisfaction was reported by 62.6% (n = 1,293) (mean ± SD: 8.74 ± 1.11), willingness to continue use by 65.6% (n = 1,356) (mean ± SD: 8.77 ± 1.11), and willingness for future procedures by 67.2% (n = 1,388) (mean ± SD: 8.84 ± 1.13). No worsening of acne was reported by 67.9% (n = 1,402) (mean ± SD: 3.28 ± 3.16).

Outcomes were consistent across all four barrier severity strata. The none/minimal stratum (n = 69) showed the highest satisfaction (mean ± SD: 9.43 ± 0.90) and lowest acne-worsening score (2.51 ± 2.95). In the moderate stratum (n = 1,172), complete dryness improvement was 57.9% (n = 679) and overall satisfaction 57.1% (n = 669). In the severe stratum (n = 223), complete dryness improvement was 62.8% (n = 140) (8.64 ± 1.14) and skin redness was 61.0% (n = 136) (8.69 ± 1.13). There were no adverse events reported during the study. These findings are shown in Figure 1.

**Figure 1:**
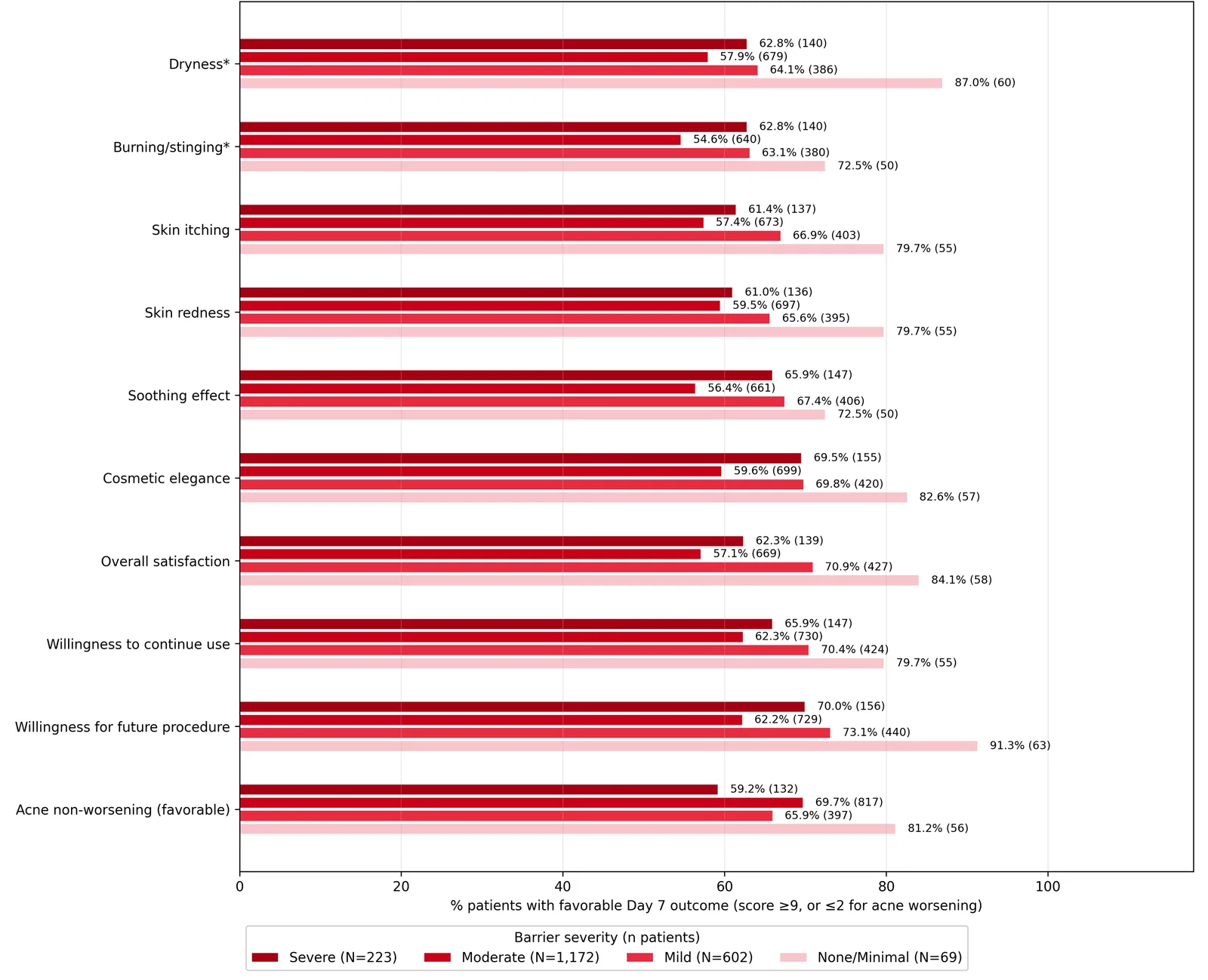
Patient-Reported Outcomes at Day 7 by Barrier Disruption Severity Stratum (N=2066) * - Primary Endpoints of Dryness and Burning/Stinging.

## Discussion

This study evaluated PROMs following the application of Episoft® AC SPF 50+, a ceramide-based moisturizer with broad-spectrum photoprotection, in 2,066 patients across 477 Indian dermatology centers after acne-related dermatological procedures. The principal finding is that patients reported substantial and rapid symptom relief by Day 7, with complete improvement achieved in the majority of patients for acute post-procedural dryness (61.2%) and burning/stinging (58.6%). Secondary outcomes closely mirrored this benefit, demonstrating robust improvement in erythema, pruritus, and soothing comfort, alongside high overall satisfaction (62.6% extreme satisfaction) and favorable adherence-proxy ratings (>65% willing to continue use and undergo future procedures). Importantly, these positive outcomes were maintained across diverse procedural modalities and across all clinician-assessed barrier disruption severity levels.

The mechanistic basis for ceramide-containing moisturizers in post-procedural care is well established. Ceramides constitute the principal structural lipid of the stratum corneum lamellar bilayer; their depletion following barrier-disrupting procedures elevates TEWL, disrupts skin hydration, and produces the subjective sensations of dryness, burning, and stinging that characterize the post-procedural recovery period [1]. Exogenous ceramide replenishment reconstitutes the lamellar lipid scaffold, accelerating barrier reconstitution. The clinical importance of early moisturization was demonstrated histologically by Collawn et al., who showed that moisturization after trichloroacetic acid (TCA) peels produced significantly more advanced epidermal repair at Day 5 versus non-moisturized controls, with reduced intercellular spacing and near-normalization of stratum corneum architecture [8]. Concurrently, acne-related procedures induce transient photosensitization that disproportionately risks PIH in the Fitzpatrick III-V skin types predominant in India, where UV and visible light exposure of barrier-disrupted skin is a well-recognized pathway to PIH and post-inflammatory erythema [3,4]. Passeron et al. demonstrated in a randomized intra-individual study that a broad-spectrum sunscreen applied to UV-exposed stripped and non-stripped skin in Fitzpatrick types IV-V improved colorimetric endpoints by 48-87% relative to unprotected controls [9]. Periprocedural skincare guidelines explicitly recommend broad-spectrum SPF 50+ in the post-procedural period [2], and both the PRACT-India and PRISM-ISF expert consensus recommendations designate photoprotection as a mandatory pillar of acne management in India [6,10]. A single formulation combining ceramide-based barrier repair with SPF 50+ thus addresses both post-procedural imperatives simultaneously - a combination that expert panels have specifically recommended to reduce regimen complexity and improve adherence [3,5].

PROMs are appropriate for evaluating post-procedural and acne-adjunct skincare products, since the parameters of clinical concern - dryness, burning, stinging, itching, erythema, cosmetic acceptability - are fundamentally subjective experiences that reflect patient comfort and drive treatment adherence. The existing PROM evidence base for combined moisturizer-plus-SPF products in acne-related indications consistently supports the present findings. In the CHARISMA study, 340 patients on stable acne therapy showed patient-reported improvement across all dermatitis parameters (itching, burning, stinging, tightness, erythema, dryness) by Day 28 with Episoft® AC, a panthenol + capric triglyceride base with SPF 30, with benefits sustained through Day 56 and high adherence [11]. Nakra et al. extended this evidence on Episoft® AC to the retinoid-induced dermatitis setting in a cohort of 1,364 patients across 357 centers, where Likert-scale PROMs demonstrated complete resolution of burning in 88.4%, stinging in 69.9%, and dryness in 70.6% at Week 4; secondary Corneometer-based hydration improved by 39.9%, providing objective corroboration of the PROM findings [12]. Kim and Kerrouche demonstrated that incorporating a moisturizer with SPF 30 into a benzoyl peroxide (BPO)-based acne regimen yielded 87% overall patient satisfaction, with 84% identifying the SPF moisturizer as a necessary element of their treatment [13]. Del Rosso et al. also showed that a combined SPF skincare regimen in adapalene-BPO therapy improved patient-reported tolerability and subject satisfaction while maintaining non-comedogenicity [14]. In a Phase IV randomized controlled trial across multiple Fitzpatrick skin types, Alexis et al. found that a combined skincare bundle including moisturizer and sunscreen alongside trifarotene significantly reduced patient-reported treatment irritation and produced meaningful PIH index reductions [15]. At the expert consensus level, the MENA Consensus explicitly highlights that combined moisturizer-SPF products may translate into better adherence than separate applications [5], consistent with evidence that optimally formulated photoprotective agents enhance adherence and prevent PIH in acne-prone skin [3,6]. The present dataset of 2,066 patients across nine procedure types constitutes the largest PROM evidence base for this product class in the acne-related post-procedural setting; it additionally captures procedure-specific PROM dimensions (willingness for future procedure, willingness to continue use) that may function as proxy adherence endpoints.

A clinically notable observation is the consistency of favorable PROMs across all four barrier disruption severity strata. Among patients with severe barrier disruption (n = 223), complete dryness improvement was 62.8% (n = 140; mean ± SD: 8.66 ± 1.08) and skin redness was 61.0% (n = 136; mean ± SD: 8.64 ± 1.11), highly comparable to the moderate stratum (57.9% [n = 679], 8.54 ± 1.12 for dryness; 59.5% [n = 697], 8.60 ± 1.08 for redness). Although Kruskal-Wallis testing identified statistically significant differences across strata (p < 0.0001), this was primarily driven by ceiling effects in the none/minimal stratum (dryness: 9.48 ± 0.80), whereas patients with moderate and severe disruption achieved comparable, substantial relief. This cross-stratum consistency indicates that combined ceramide-SPF formulations may provide meaningful patient-reported benefit across both superficial and deeper procedural interventions.

A nuanced interpretation is warranted for the acne worsening PROM endpoint. While 67.9% reported no or minimal worsening (score 1-2; mean ± SD: 3.28 ± 3.16), 13.0% reported high worsening (9-10). Chemical peels, comprising more than half of the procedures included in this study, exert their therapeutic effect through controlled keratolytic exfoliation of the epidermis. This desquamative process can transiently increase acne lesion counts during the exfoliation phase, a recognized consequence of peel-induced skin turnover that can be independent of any post-procedural product applied [16,17]. Notably, acne flare-ups are a recognized adverse event following chemical peels; in a randomized controlled trial in 40 acne patients, comparing 20% salicylic acid plus 10% mandelic acid peel with 35% glycolic acid peel, acne flare-up was reported in 10% of patients in both treatment arms, while visible desquamation occurred in 80% and 40% of patients, respectively [17]. A 67.9% non-worsening rate in a cohort where the majority underwent a procedure intrinsically associated with transient acne flares represents a clinically acceptable PROM tolerability profile. Prospective studies incorporating pre- and post-procedure acne severity scoring would enable more precise attribution of worsening to procedural versus product factors.

This study has several limitations that define the boundaries of inference. The observational design without a comparator arm precludes definitive causal attribution of symptom relief to Episoft® AC SPF 50+; without an untreated or vehicle control group, the natural resolution of post-procedural symptoms cannot be separated from product-specific effects. The reliance on patient-reported numeric rating scales, while methodologically appropriate for capturing subjective recovery and comfort, was not paired with objective biophysical assessments (e.g., TEWL or Corneometer hydration). Although data compilation was retrospective, PROMs were completed prospectively by patients at their Day 7 clinic visit, restricting recall to the preceding week; nonetheless, seven-day recall and potential social desirability bias during in-clinic questionnaire completion (which may contribute to the low frequency of 'none' improvement ratings) remain recognized limitations. The absence of pre-procedural baseline symptom scores precludes calculation of mathematical change scores. Furthermore, pragmatic inclusion criteria without restrictive exclusion parameters introduce clinical heterogeneity regarding baseline acne severity, specific device parameters, and concomitant medications. The dominance of chemical peels (56.7%) and the lack of individual Fitzpatrick phototype recording also limit subgroup generalizability. These limitations underscore the need for prospective, randomized, vehicle-controlled trials combining PROMs with objective biophysical and photopigmentary endpoints.

## Conclusions

In this large, real-world multicenter study conducted across 477 Indian dermatology centers, Episoft® AC SPF 50+, a ceramide-based moisturizer with SPF 50+, was associated with substantial patient-reported symptom relief, high satisfaction, and minimal acne worsening at Day 7 following diverse acne-related dermatological procedures. Complete improvement was reported by over 58% of patients for both primary PROM endpoints, with favorable and consistent outcomes across all four barrier disruption severity levels and nine procedure types. These findings, situated within a converging body of PROM-based evidence for combined moisturizer-plus-SPF products, support the routine inclusion of ceramide-based SPF 50+ moisturization in post-procedural skincare regimens for acne-related procedures.

## Figures and Tables

**Figure 2:**
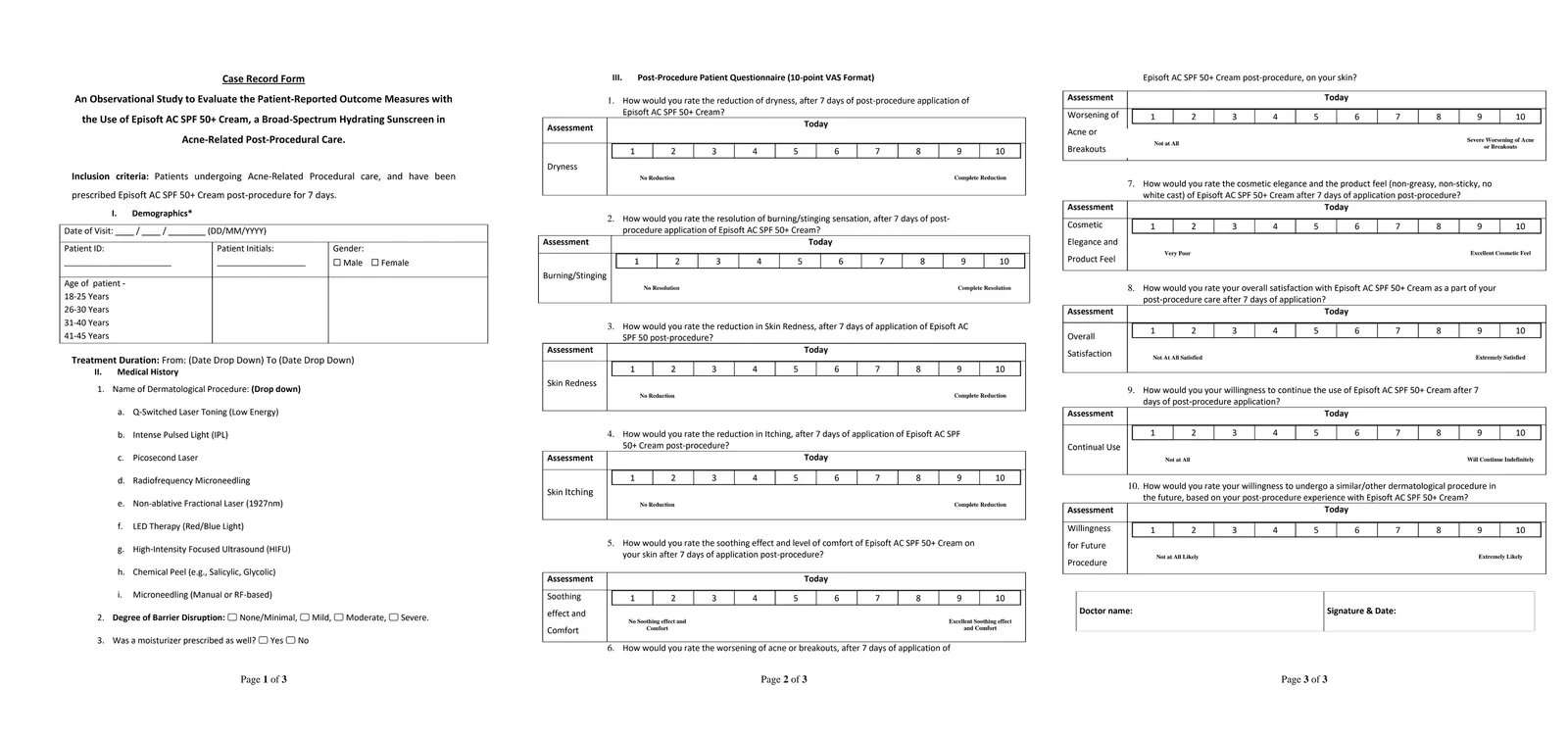
Case Record Form Abbreviations: IPL, intense pulsed light; LED, light-emitting diode; VAS, visual analogue scale.

## Appendices

Appendix 1 

**Table 3:** Distribution of Post-procedural Skin Barrier Disruption Across Dermatological Procedures (N = 2,066) Post-procedural Skin Barrier Disruption Classification by Procedure Type (Table 1) was used; additionally, barrier disruption level was also independently evaluated and categorized by treating dermatologists for each patient based on procedure intensity, depth of injury, energy density/fluence, number of passes, acid concentration/peel depth, and observed immediate cutaneous response. Row percentages represent the proportion of patients within each procedure assigned to that severity stratum.

Procedure Type	None / Minimal, n (%)	Mild, n (%)	Moderate, n (%)	Severe, n (%)	Total Cohort, n (%)
Chemical Peel (salicylic/glycolic acid)	29 (2.5)	343 (29.3)	710 (60.6)	90 (7.7)	1,172 (56.7)
Q-Switched Laser Toning (low energy)	23 (10.2)	100 (44.2)	81 (35.8)	22 (9.7)	226 (10.9)
Microneedling (Manual)	4 (2.2)	54 (29.5)	102 (55.7)	23 (12.6)	183 (8.9)
Radiofrequency Microneedling	4 (3.7)	18 (16.7)	68 (63.0)	18 (16.7)	108 (5.2)
LED Therapy (Red/Blue Light)	3 (3.0)	23 (22.8)	53 (52.5)	22 (21.8)	101 (4.9)
Picosecond Laser	1 (1.1)	22 (23.7)	60 (64.5)	10 (10.8)	93 (4.5)
Non-ablative Fractional Laser (1,927 nm)	0 (0.0)	20 (23.8)	48 (57.1)	16 (19.0)	84 (4.1)
Intense Pulsed Light (IPL)	3 (5.9)	17 (33.3)	23 (45.1)	8 (15.7)	51 (2.5)
High-Intensity Focused Ultrasound (HIFU)	2 (4.2)	5 (10.4)	27 (56.2)	14 (29.2)	48 (2.3)
Total Cohort	69 (3.3)	602 (29.1)	1,172 (56.7)	223 (10.8)	2,066 (100.0)

Appendix 2

## References

[1] Rajkumar J, Chandan N, Lio P, Shi V (2023). The skin barrier and moisturization: function, disruption, and mechanisms of repair. Skin Pharmacol Physiol.

[2] Goodman G, Yip L, McDonald C, Lin F, Liu W, Sullivan J (2025). Recommendations on periprocedural skincare for energy-based dermatologic procedures. Aesthet Surg J Open Forum.

[3] Piquero-Casals J, Morgado-Carrasco D, Rozas-Muñoz E (2023). Sun exposure, a relevant exposome factor in acne patients and how photoprotection can improve outcomes. J Cosmet Dermatol.

[4] Lim HW, Zhang C, Taylor M (2026). International expert consensus on knowledge gaps in care for Dermatologic disorders in skin of color. Int J Dermatol.

[5] Zimmo S, Zaher H, Said A (2026). Use of dermocosmetics in acne management: a Middle East-North Africa consensus. Int J Dermatol.

[6] Madnani N, Saraswat A, Nott A (2025). PRACT-India: practical recommendations on acne care and medical treatment in India-a modified Delphi consensus. Antibiotics (Basel).

[7] (2019). New drugs and clinical trials rules, 2019. https://cdsco.gov.in/opencms/opencms/en/Acts-and-rules/New-Drugs/.

[8] Collawn SS, Boissy RE, Gamboa M, Vasconez LO (1998). Ultrastructural study of the skin after facial chemical peels and the effect of moisturization on wound healing. Plast Reconstr Surg.

[9] Passeron T, Brown A, Furmanczyk M, Foyaca M, Trullas C, Piquero-Casals J (2026). An investigator-blinded, randomized trial of a broad-spectrum sunscreen containing sclareolide and niacinamide for the prevention of post-inflammatory hyperpigmentation in skin of color. Dermatol Ther (Heidelb).

[10] Kohli M, Srinivas CR, Saraswat A (2025). Practical recommendations for Indians on sunscreen use-a modified Delphi consensus by Indian Sunscreen Forum (PRISM-ISF). J Cosmet Dermatol.

[11] Bhat SA, Deshmukh GA, Dhoot DS, Barkate H (2018). Comprehensive skin care regimen of moisturizer with broad-spectrum sunscreen as an adjuvant in management of acne (CHARISMA). Int J Sci Stud.

[12] Nakra G, Modi A, Hegde S (2026). Real-world evaluation of the Episoft® AC moisturizer with broad-spectrum sunscreen in the management of retinoid-induced dermatitis: a retrospective observational study. Cureus.

[13] Kim MR, Kerrouche N (2018). Combination of benzoyl peroxide 5% gel with liquid cleanser and moisturizer SPF 30 in acne treatment results in high levels of subject satisfaction, good adherence and favorable tolerability. J Dermatolog Treat.

[14] Del Rosso JQ, Gold M, Rueda MJ, Brandt S, Winkelman WJ (2015). Efficacy, safety, and subject satisfaction of a specified skin care regimen to cleanse, medicate, moisturize, and protect the skin of patients under treatment for acne vulgaris. J Clin Aesthet Dermatol.

[15] Alexis A, Del Rosso JQ, Forman S (2024). Importance of treating acne sequelae in skin of color: 6-month phase IV study of trifarotene with an appropriate skincare routine including UV protection in acne-induced post-inflammatory hyperpigmentation. Int J Dermatol.

[16] Kontochristopoulos G, Platsidaki E (2017). Chemical peels in active acne and acne scars. Clin Dermatol.

[17] Chen X, Wang S, Yang M, Li L (2018). Chemical peels for acne vulgaris: a systematic review of randomised controlled trials. BMJ Open.

